# Novel Hydrophilic Oligomer-Crosslinked Gelatin-Based Hydrogels for Biomedical Applications

**DOI:** 10.3390/gels9070564

**Published:** 2023-07-11

**Authors:** Mamoona Tariq, Rabia Khokhar, Arslan Javed, Muhammad Usman, Syed Muhammad Muneeb Anjum, Huma Rasheed, Nadeem Irfan Bukhari, Chao Yan, Hafiz Awais Nawaz

**Affiliations:** 1 School of Pharmacy, Shanghai Jiao Tong University, Dongchuan Road 800, Minhang District, Shanghai 200240, China; mt14@sjtu.edu.cn (M.T.);; 2 Punjab University College of Pharmacy (PUCP), University of the Punjab, Lahore 54000, Pakistan; 3 Institute of Pharmaceutical Sciences (IPS), University of Veterinary & Animal Sciences (UVAS), Lahore 54000, Pakistan

**Keywords:** hydrogels, free radical polymerization, gel permeation chromatography (GPC), nuclear magnetic resonance (NMR), rheology, biomedical applications

## Abstract

Gelatin-based hydrogels have shown good injectability and biocompatibility and have been broadly used for drug delivery and tissue regeneration. However, their low mechanical strengths and fast degradation rates must be modified for long-term implantation applications. With an aim to develop mechanically stable hydrogels, reactive anhydride-based oligomers were developed and used to fabricate gelatin-based crosslinked hydrogels in this study. A cascade of hydrophilic oligomers containing reactive anhydride groups was synthesized by free radical polymerization. These oligomers varied in degree of reactivity, comonomer composition, and showed low molecular weights (M_n_ < 5 kDa). The reactive oligomers were utilized to fabricate hydrogels that differed in their mechanical strengths and degradation profiles. These formulations exhibited good cytocompatibility with human adipose tissue-derived stem cells (hADCs). In conclusion, the reactive MA-containing oligomers were successfully synthesized and utilized for the development of oligomer-crosslinked hydrogels. Such oligomer-crosslinked gelatin-based hydrogels hold promise as drug or cell carriers in various biomedical applications.

## 1. Introduction

Hydrogels are a special class of biocompatible, 3D, polymeric materials that can function as scaffolds and imitate the characteristics of different bodily tissues. They have been incredibly popular in recent years as a result of their capacity to sustain high water content, keep a porous structure, and adapt to various sol-gel conditions [1]. The physicochemical characteristics of these hydrogels have been modified over time by researchers to create “smart” or “intelligent” hydrogels that respond to environmental factors such as temperature, acid levels, light, magnetic and electric fields, ionic strength, or the enzymatic environment [2,3]. Such hydrogels have demonstrated significant promise in non-invasive, remote-controlled therapies, including targeted drug administration, prosthetics, implant coatings, wound healing, regenerative medication, tissue engineering, and artificial organ implantation [1,4,5]. Several therapeutic agents can be encapsulated into hydrogels, which can adapt to any shape of the defect and surrounding tissue. The use of injectable hydrogels in gene therapy, controlled delivery of medications, cells, and growth factors, nucleus pulposus replacement, cardiac repair, and regenerative uses for cartilage and bone have increased recently [6,7,8,9,10]. Fundamental research using embryonic stem cells and induced pluripotent stem cells has also been actively conducted to establish this revolutionary treatment in regenerative medicine [11]. Tissue engineering therapies are also becoming more significant with an eye toward clinical applications [12].

Hydrogel systems are categorized into two major classes: physical crosslinking systems, such as those based on charge attractions, hydrophobic interactions, and stereo complexation, and chemical crosslinking systems using the Michael addition reaction and UV irradiation [13,14]. In general, the in situ-produced gel’s mechanical integrity is maintained and controlled via a chemical crosslinking procedure [15]. For chemical crosslinking, polymerizable moieties and distinct functional groups are required. From a hydrogel design standpoint, it is preferable that these conjugation events, which are also referred to as bio-orthogonal reactions, advance with high specificity, good conversion rates, and indifference to the surrounding biological system [16]. Improved and cost-effective strategies are required to fabricate a hydrogel system with such parameters.

Numerous in situ-forming hydrogels have been explored over the last few decades. In situ, crosslinkable hybrid hydrogels comprising gelatin and 4-arm polypropylene oxide–polyethylene oxide (Tetronic) were synthesized as injectable scaffolds for tissue regeneration [14]. Moreover, injectable formulations utilizing combinations of hydrophilic comonomers hydroxypropyl acrylate (HPA), acryloyl morpholine (AMO), N-vinyl pyrrolidone (NVP), hydrophobic pentaerythritol diacrylate monostearate (PEDAS), and reactive maleic anhydride (MA) were also synthesized [8]. 

In the field of polymer chemistry, oligomers occupy a distinct intermediate chemical space between discrete small molecules and high molecular weight polymers [17]. Inspiration from macromolecular chemistry and small molecule organic chemistry guides the synthetic approaches for preparing oligomers. Their properties may vary significantly by changing one or more units in their composition [18]. Controlled composition and intermediate molecular weights are key factors in optimizing materials for future applications. Inspired by the aforementioned advantages, the current research was designed to develop novel oligomers through a free radical polymerization method that were reactive, possessed appropriate molecular weights and controlled comonomer compositions, and could be utilized to fabricate oligomer-crosslinked hydrogels with appropriate stiffness values. Such reactive oligomers based on the presence of anhydride moieties had good biocompatibility along with unique degradation properties and structural versatility. Different variations of comonomer types and ratios were used to achieve the desired oligomers. While preparing the hydrogels, anhydride groups of oligomers could covalently crosslink with amine-containing molecules, i.e., gelatin. The resultant hydrogels showed favorable properties and composition-dependent storage modulus values with stromal stem cell encapsulation capability.

## 2. Results and Discussion

### 2.1. Synthesis of Two Novel Sets of Oligomers, i.e., oligo LA-co-HEA-co-MA (oLHM), oligo LA-co-HEMA-co-MA (oLHeM), and oligo SA-co-HEA-co-MA (oSHM), oligo SA-co-HEMA-co-MA (oSHeM)

The strategy of designing hydrogels from reactive anhydride-containing oligomers and gelatinous polypeptides is fascinating and can enable the fabrication of hydrogels with a range of mechanical strengths for a variety of applications. Anhydride–amine-based crosslinking is captivating as the anhydrides exhibit good reactivity but hydrolyze on exposure to the aqueous environment without posing any hazard to the physiological system, a remarkable advantage over conventional crosslinkers such as glutaraldehyde. In the last decade, the research group of Dr. Hacker has published phenomenal research on reactive anhydride-containing oligomers. These include thermosensitive N-isopropyl acrylamide (NiPAAm), reactive pentaerythritol diacrylate monostearate (PEDAS)-containing oligomer (oPNMA) [19,20,21,22,23], and diacetone acrylamide-based oligomer (oPDMA) [24,25] as injectable hydrophilic oligomers [8,26] for applications in drug/cell delivery, tissue engineering, sustained release of siRNA, covalent modification of aECM, etc. However, in the current study, hydrophobic domaincontaining hydrophilic comonomers were altered to delineate their effects on the mechanical properties of oligomer-crosslinked hydrogels. 

The reactive oligomers were designed to contain a hydrophobic domain, either stearyl acrylate (SA) or lauryl acrylate (LA), to stabilize the hydrogels [27]. Hydroxyl group-containing hydrophilic comonomers such as hydroxyethyl acrylate (HEA) and hydroxyethyl methacrylate (HEMA) were tested as comonomers, which provided hydrophilicity and a number of available hydroxyl groups for further chemical modification, if required [21,28]. The design criterion was maintained as a 1:20 ratio of hydrophobic domain: sum of other comonomers, as previously published [8,19,20,21,22,23]. Subsequently, two sets of reactive ter-oligomers (having three comonomers each) were synthesized, which differed in the type of hydrophobic domain (SA or LA), comonomer type (HEA or HEMA), and comonomer to maleic anhydride ratio to meet the design criteria of 1:20 (Table 1 and Table 2). Both HEA [26] and HEMA [28] have previously been tested for their biomedical applications.

### 2.2. Characterization of Oligomers

**Figure 1:**
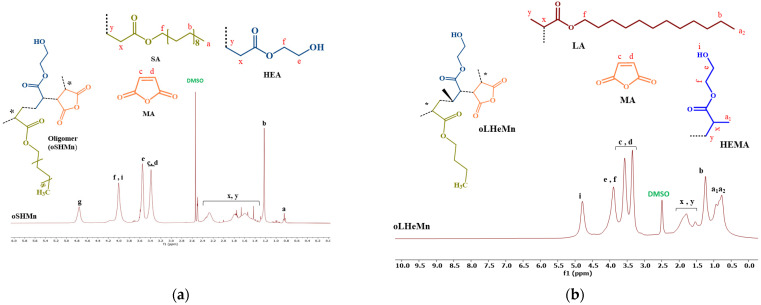
^1^H-NMR spectra of (**a**) oSHM and (**b**) oLHeM oligomers. Specific protons in comonomers have been illustrated using different letters. Asterisks (*) indicate further polymerization.

**Figure 2:**
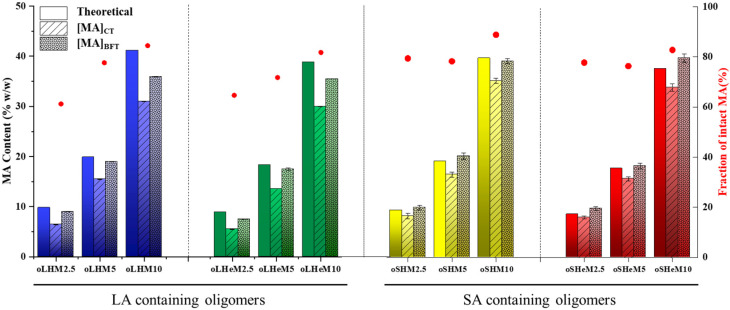
MA contents of ter-oligomers (left y-axis) based on the comonomer feed (bars without any pattern, theoretical), oligomer-integrated MA contents (bars with oblique striations, [MA]_CT_), total intact anhydride moiety contents (bars with filled dots, [MA]_BFT_), and fraction of intact MA units (right y-axis, indicated by red dots). Columns and error bars indicate averages and standard deviations. All experiments were performed in triplicate.

Conductometric and Brown–Fujimori titrations were performed for the assessment of MA integration and intactness in both groups of ter-oligomers [8,19]. Contrary to the other comonomers (SA, LA, HEA, and HEMA), incorporation of MA in the ter-oligomers could not be quantified using ^1^H-NMR owing to overlapping signals, as shown in Figure 1a,b. Figure 2 exhibits the relationship between the theoretical incorporation of MA units (measured by comonomer feed), total percentage of MA content in the ter-oligomer (measured by conductometric titration), and intact anhydride moiety contents (measured by Brown–Fujimori titration).

Overall, MA input controlled the anhydride content in all synthesized ter-oligomers. A continuously increasing but non-linear relationship was found between MA incorporation [MA]_CT_ and theoretic input. With low MA feed (MA = 2.5), the theoretical input approximately matched the [MA]_CT_. For higher MA feeds (MA = 5 and 10), the [MA]_CT_ was always lower than the MA feed. The molar concentration of MA units in comparison to the hydrophobic domains (SA or LA) is summarized in Table 3 for both groups of ter-oligomers. A fraction of the MA units might have hydrolyzed during the synthesis, purification, and/or storage procedures, as exhibited by higher values of [MA]_BFT_ than [MA]_CT_ for all ter-oligomers. The fraction of intact MA units always ranged between 75% and 90%, indicating the controlled procedure for anhydride synthesis.

Among the synthesized groups of ter-oligomers, an MA feed of 5 was determined as appropriate. Therefore, all oligomers were synthesized using similar ratios along with lower (2.5) and higher (5) MA feeds. ^1^H-NMR and ^13^C-NMR spectral analysis confirmed the successful incorporation of the constituting comonomer in all oligomers (Figure 1, and Figure S3 in supplementary materials). Generally, incorporation of the hydrophilic comonomer and MA units remained under control. However, incorporation of HEA and HEMA was better controlled in SA-containing oligomers (Table 3). MA incorporation was consistent with the previously published data for oPNMA and oPDMA oligomers [19,24].

The reactivity of the oligomers was investigated by conductometric titrations (MA integration) and Brown–Fujimori titrations (MA intactness) [19,29]. Figure 2 illustrates the relationship between the MA content in the theoretical feed, the MA incorporated in the oligomer, and total intact anhydride moieties for the LA- and SA-containing ter-oligomers. Overall, MA incorporation in all ter-oligomers was dependent upon the theoretical MA feed. A strictly increasing but non-linear relationship was found between the MA theoretical feed and oligomer MA incorporation. With low MA feed (MA = 2.5), values for [MA]_CT_ and the theoretical feed were very close to each other. The values were higher at higher MA feed (MA = 5 or 10) for LA-containing ter-oligomers. However, SA-containing oligomers exhibited good control over MA incorporation ([MA]_CT_) in relation to the MA theoretical feed. In all cases, [MA]_BFT_ illustrated higher values, which could be due to a fraction of the MA content being hydrolyzed during synthesis, storage, or analysis. In addition, 60–90% of the anhydride units were calculated to be preserved for all ter-oligomers, an indication of good reactivity of the oligomers for further crosslinking reactions [22,24].

The synthesis protocol resulted in the formation of oligomers with low molecular weights (M_n_ < 5 kDa, Table 4). Generally, the oligomers exhibited a decrease in molecular weight with an increase in the number of MA units. This showed the preference for MA polymerization as the MA theoretical input increased. GPC analysis revealed controlled molecular weights in the range of 1.81 to 3.76 kDa (M_n_ values) for all types of oligomers. Generally, the molecular weights for oLXMs were smaller than the molecular weights of oSXMs. However, all of the values correlated well with the theoretical calculation of molecular weights for these oligomers and the previous literature [21].

**Figure 3:**
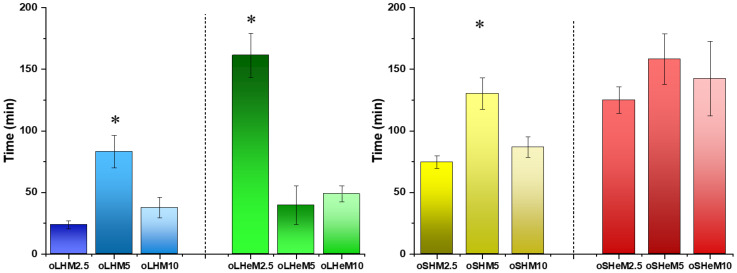
Dissolution studies on two sets of hydrophilic oligomers (oSXMs and oLXMs) for different MA feeds. The bars indicate the time at which 60% of maximum light transmittance was achieved as a measure of initial dissolution kinetics. Asterisks (*) indicate significant differences with other members of the same group. All experiments have been performed in triplicate.

The hydrophilicity of the oligomers was compared after measuring their water dissolution kinetics. During the dissolution evaluation, the anhydride moieties will be hydrolyzed, thereby changing the oligomer structures to make them more water soluble. However, the first contact between the newly synthesized oligomers with water decides the initial dissolution rate. The study results are illustrated in Figure 3. Generally, all oligomers exhibited fast dissolution rates in comparison to previously published data [8]. Lauryl acrylate (C_12_)-containing oligomers (oLXMs) showed faster dissolution rates in comparison to the oligomers (oSXMs) containing stearyl acrylate (C_18_) as the comonomer. Within each group (oLXM or oSXM), the HEMA-containing oligomers showed delayed dissolution rates in comparison to the HEA-containing oligomers. However, the absence of a significant difference prevented reaching an obvious conclusion. Delayed dissolution rates for the oSXM oligomers in comparison to oLXM oligomers could be attributed to the longer hydrophobic domain, i.e., stearyl acrylate (C_18_), in these oligomers. Polymers with different hydrophobic domains have shown promising results in cell internalization [30] and gene delivery [31] studies in the past. 

**Scheme 1:**
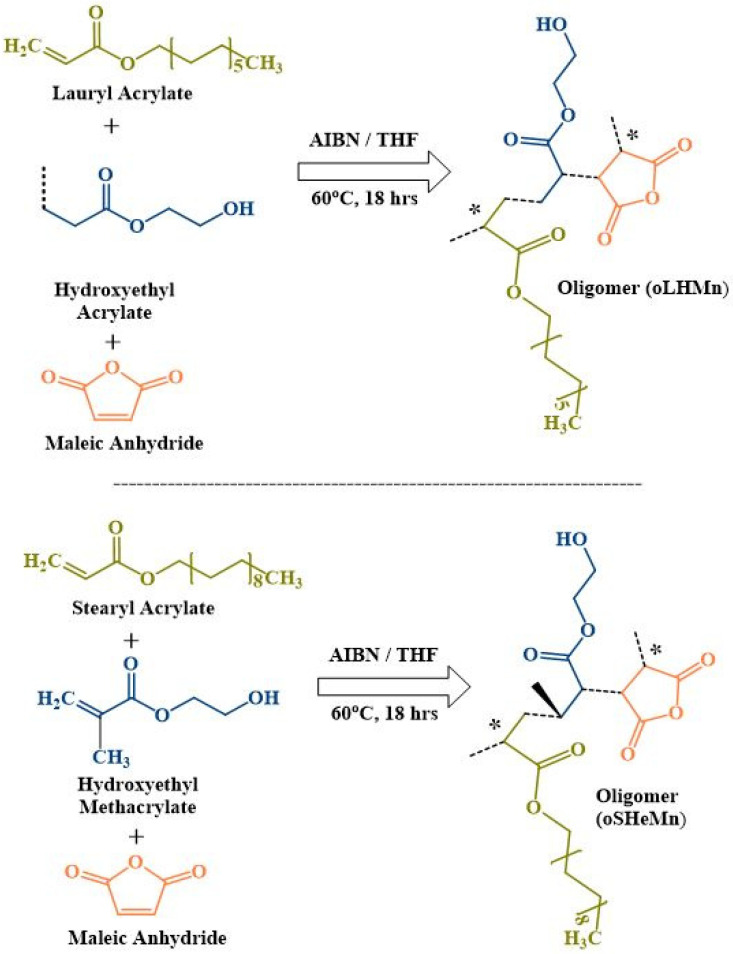
Oligomer (oLHMn) and (oSHeMn) synthesis reaction. Synthesized oligomer (*oligo* LA-*co*-HEMA-*co*-MA or *oligo* SA-*co*-HEA-*co*-MA)**,** hydrophobic domain (LA), a monomer with desired properties (HEMA), and reactive monomer (MA) reacted together through free radical polymerization to give a hydrophilic oligomer. Asterisks (*) represent the possible points for branching of the oligomer.

In these pristine hydrophilic oligomers, the anhydride moieties were well-preserved during the synthesis, purification, and storage conditions, and could be used for anchoring or crosslinking reactions (Scheme 1). Among typical nucleophiles, many amines and alcohols have been investigated before [32]. Due to the possibility of using amines at low temperature [33] in a buffered environment and the established record of anhydride–amine conjugation in many biomedical applications [1,34], amines were used for crosslinking the pristine oligomers. The presence of intact amines is the prerequisite for the anhydride–amine reaction; thus, a pH-controlled or buffered environment can facilitate the reaction. Collagen, as a structural component of the extracellular matrix (ECM), or its partial hydrolysate, has been used previously for this reaction [20,22,23,24,25,35]. The protein not only provides amine groups but also imparts biological functionality to the crosslinked matrix. Collagen-derived peptides can mimic a physiological ECM-like environment that motivates cell signaling (due to the presence of integrin-binding domains) and cell adhesion (owing to the presence of RGD (cell adhesive) motifs) [36,37]. In addition, collagen-derived gelatin molecules have shown advanced properties such as being antigen-free and non-immunogenic. Previously, gelatin with lower bloom values (G50 and G160) have also been investigated for amine–anhydride conjugation [20]. However, owing to better gelation and cytocompatibility results (data not shown here), higher bloom value gelatin (G300) was utilized in the current investigation. 

The pristine hydrophilic oligomers presented inherent reactivity owing to the presence of intact anhydride moieties. As an aqueous solution of the oligomer could hydrolyze the anhydride units, the oligomers were dissolved in the minimum possible quantity of organic solvent mix (DMSO and NMP). The quantities of oligomer and organic solvent mix were set in a way to keep the oligomer in dissolved form without affecting the gelation and cytocompatibility properties. Such an oligomer organic solution could be mixed with an aqueous solution of gelatin, in the presence of an aqueous base solution, to facilitate amine–anhydride conjugation. For the fabrication of homogenous crosslinked matrices, appropriate quantities of each component were identified in preliminary experiments. The published data also confirmed the cytocompatibility of the organic solvents [8]. Among the base molecules, NMPO was selected because it facilitated homogenous gelation and showed better cytocompatibility results (data not shown here). 

After a thorough adjustment and selection of the oligomer solvent, base molecule, and gelatin type, the three solutions were mixed to fabricate the gelatin-crosslinked hydrogels by amine–anhydride conjugation (Scheme 1). The fabricated hydrogels comprised three solutions: (1) an oligomer solution in organic solvent mix (DMSO and NMP), (2) an aqueous solution of base (NMPO), and (3) a gelation solution prepared in cell culture medium (DMEM). Oscillation rheology was utilized to monitor the crosslinking reactions for these hydrogels. 

**Figure 4:**
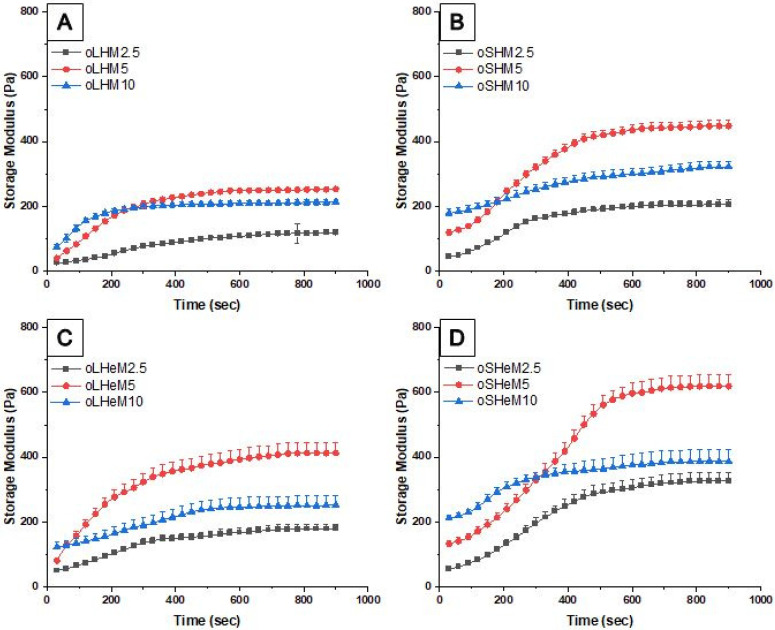
Rheological characterization of crosslinking reactions between gelatin and different oligomers. Time sweep profiles of storage modulus values (G′) for different oligomers: (**A**) oLHM, (**B**) oSHM, (**C**) oLHeM, and (**D**) oSHeM. Means and standard deviations (*n* = 3) have been represented by data points and error bars.

Hydrogel mechanics play a crucial role in determining applications in the fields of drug delivery, tissue engineering, and regenerative medicine. Noteworthy parameters include the diffusion potential, differentiation priority of progenitor cells, and tissue integration [38,39]. Oscillation rheology was utilized to monitor the crosslinking reactions for these hydrogels and the results are summarized in Figure 4. Generally, within each group, the MA-2.5-containing oligomer-crosslinked hydrogels showed the highest achieved storage modulus values (G′). The crosslinked hydrogels fabricated with lower MA-containing oligomers (2.5) showed lower storage modulus values, which was attributed to a lower number of MA units and thus a decreased number of crosslinks developing in these hydrogel systems. On the other hand, the MA-4.5-containing oligomer-crosslinked hydrogels did not exhibit higher storage modulus values. This could be due to the fast gelation reaction between the higher numbers of anhydrides and gelatin chains. Initial fast gelation can obstruct further gelation reactions due to steric hindrance or gelatin chain immobility, thereby causing the loss of anhydride moieties without being utilized in crosslinking reactions. 

**Figure 5:**
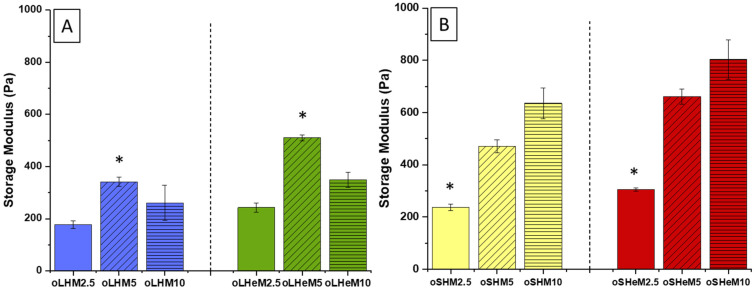
Storage modulus values for pre-fabricated hydrogels from oLXM (**A**) and oSXM (**B**) oligomers with different hydrophobic domains and MA contents. Average and standard deviation values (*n* = 5) are represented by columns and error bars. Asterisks (*) indicate statistically significant differences (*p* < 0.05).

Pre-fabricated hydrogels were formulated in polypropylene molds by mixing the oligomer and gelatin solutions in a buffered environment. The molds were incubated in tissue culture medium overnight before performing the rheological characterization. Subsequently, the storage modulus values of the hydrogels were measured using a rheometer. All of the hydrogels presented complete gelation and well-formed structures. Also, no gel deformation was observed during the time sweep characterization of these hydrogels. Generally, the results revealed the dependance of the storage modulus value on the oligomer composition, comonomer composition, and number of anhydride moieties in the oligomer composition. Within each group, the MA-5-containing oligomer-crosslinked hydrogels showed the highest storage modulus values in comparison to the MA-2.5- and MA-10-containing oligomer-crosslinked hydrogels (Figure 5). The results explicitly exhibited the significance of adjusting the amounts of anhydride moieties, comonomer ratios, and composition of oligomers for fabricating oligomer-crosslinked hydrogels. 

The storage modulus values were affected by the quantities of anhydride units. Within all groups, the MA-5-containing oligomer-crosslinked hydrogels presented stiffer hydrogels in comparison to the hydrogels fabricated with MA-2.5-containing oligomers. In the oLXM group, the HEA-containing oligomers presented weaker hydrogels in comparison to the HEMA-containing oligomers. A similar trend was also observed in the oSXM oligomer-crosslinked hydrogels. The general trend in storage modulus values was observed as oLHM < oLHeM < oSHM < oSHe. The MA-5-containing oligomer-crosslinked hydrogels clearly revealed statistically significant differences. However, statistically significant differences could not be found for the MA-2.5- and MA-7.5-containing oligomer-crosslinked hydrogels. In the oSXM group, an increasing trend of storage modulus values was observed (oSHM-2.5 < oSHeM-2.5 < oSHM-5 < oSHM-7.5 < oSHeM-5 < oSHeM-7.5). Weaker hydrogels fabricated with MA-2.5-containing oligomers due to an insufficient number of anhydride moieties and/or too fast or inconsistent crosslinking with MA-7.5-containing oligomers could have been the reason for not showing statistically significant differences. These differences in storage modulus values could be clearly attributed to the composition of the hydrogels [40,41,42] and have been under investigation for different cell culture-based studies. The hydrogels exhibited comparable storage modulus values to the stiffness of soft body tissues [43] and could be useful for conducting research experiments in tissue engineering [44]. Previous studies with similar stiffness values have demonstrated the survival, spreading, and growth of neurites [43,45] and adipose tissue-derived stem cells [46] in hydrogels with similar storage modulus values. These results demonstrated that the composition of these tunable materials influences their mechanical properties [47]. This also relates to their degradation rates, which is relevant for their applications as biomaterials [40].

**Figure 6:**
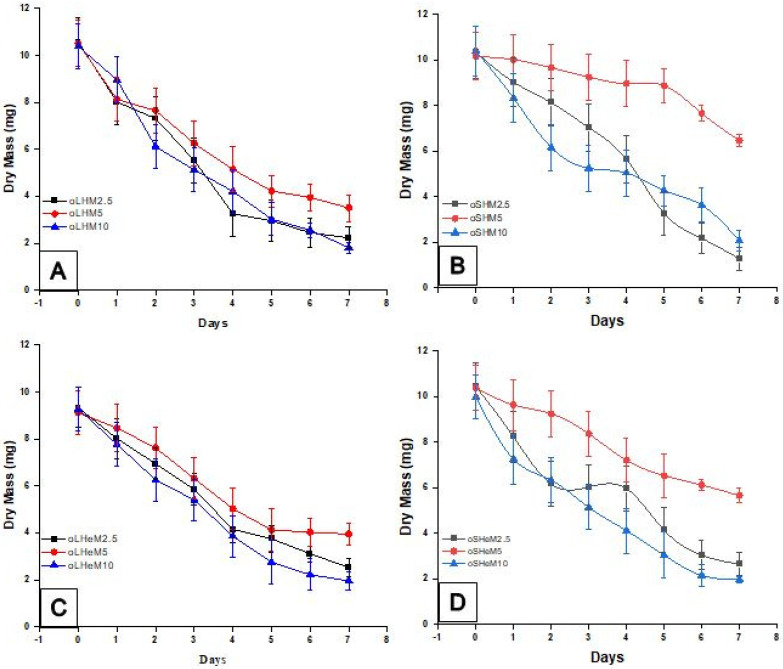
Fluctuations in pH values of all three different groups of oLHM (**A**), oSHM (**B**), oLHeM (**C**), and oSHeM (**D**) hydrogel formulations during hydrolytic degradation in PBS at 37 °C. Dotted line at pH 7.4 shows the ideal pH to which the pH of all oligomers was compared.

Figure 6 elucidates the dry weights of the oligomer-crosslinked hydrogels for all compositions. The dry weights were determined gravimetrically. Determination of dry masses over a period of 2 weeks indicated that the crosslinker composition was the most important for holding the gelatin molecules together. Generally, the crosslinked hydrogels for all groups exhibited smooth degradation profiles in the stipulated time. The minor rise in the dry weights at early time points could be attributed to the deposition of salts and residual water in the hydrogels. Overall, the MA-5-containing oligomers were the most stable hydrogels in all groups. This could be related to the uniform and complete crosslinking in these oligomer-based hydrogels. On the other hand, insufficient crosslinking in the MA-2.5-containing crosslinked hydrogels and inconsistent crosslinking in the MA-7.5-containing crosslinked hydrogels led to the formation of loose and inhomogeneous matrices that showed faster degradation rates in the stipulated time. Comparing the oligomers, the oLHM crosslinked hydrogels showed the fastest degradation rates in PBS and less than 30% intact mass remained after 2 weeks. In comparison, the oLHeM and oSHM crosslinked hydrogels exhibited more than 30% dry mass after a similar duration. However, the oSHeM crosslinked hydrogels showed delayed degradation and only 50% mass was lost in 2 weeks. The resilience to degradation for the oSHeM crosslinked hydrogels could be linked to the high storage modulus values of these matrices (Figure 6). Oligomer composition, homogeneity, and maximum crosslinking resulted in delayed degradation for these matrices. Insignificant variations in pH values were observed in the degrading solutions. Quick degradation was observed after 2 weeks and no substantial residues were observed. In comparison to this *in vitro* degradation profile, enzymatic activity can lead to faster degradation for gelatin-based matrices *in vivo* [23]. 

The MA-5-containing oligomer-crosslinked hydrogels from all groups were fabricated with encapsulated adipose tissue-derived stromal cells (ADCs), as previously described, and evaluated for cytocompatibility and proliferation using the WST-1 assay on days 1, 3, and 7. For this purpose, the hydrogels were prepared in polypropylene molds and maintained in 24-well plates in cell culture medium. Such empty molds were used as controls to investigate cell interaction and proliferation on the surface of these molds. No cell proliferation could be found on these empty molds (data not shown). Therefore, cell spreading and proliferation could be attributed to cell–hydrogel interplay. To compare the growth of cells encapsulated in the hydrogels, cells were seeded on PS empty molds and incubated in cell culture medium. 

**Figure 7:**
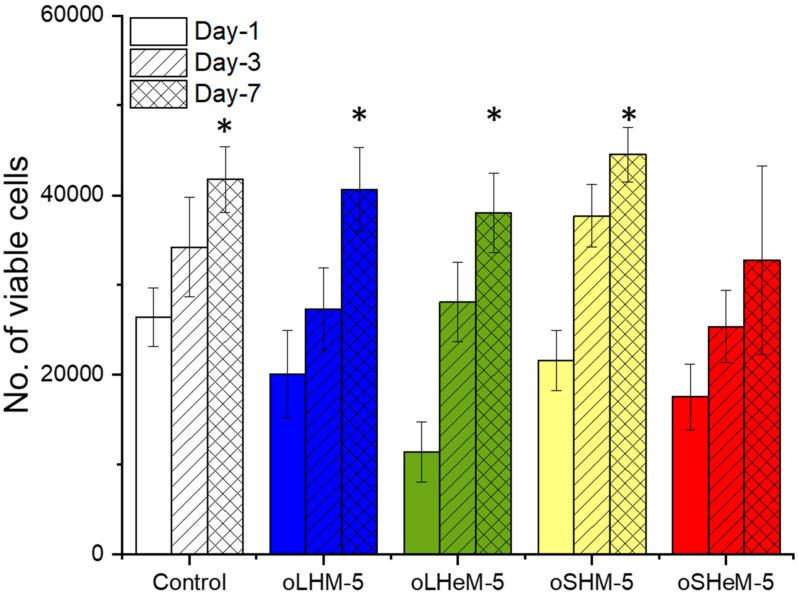
Comparison of cell viability at days 1, 3, and 7. Asterisks (*) indicate significant cell proliferation in comparison to cell number on day-1.

As expected, all of the hydrogel groups presented time-dependent cell proliferation, which was parallel to the 2D cell proliferation on PS and highest on day 7 (Figure 7). In all groups, cell proliferation on day 7 was significantly increased in comparison to the cell count on day 1 in the same group. The oSHeM crosslinked hydrogels, however, did not show significantly different results. This could be due to the high standard deviation values found on day 7 for these hydrogels. It is believed that all cells show equal metabolic activity in the absence of any differentiation stimuli. Therefore, the different cell numbers were attributed to variations in metabolic activity. Some amount of tetrazolium salts could have also been adsorbed on the hydrogel surface, making it unavailable to the cells for their metabolic activity. Cell morphology was found to be round on day 1, while elongated shapes were observed on days 3 and 7 (data not shown here). Material–cell interaction is an important characteristic for biomedical applications [48]. The enhanced cell–hydrogel interaction could be attributed to the natural gelatin biocompatibility [49].

## 3. Conclusions

Four sets of reactive and cytocompatible ter-oligomers were synthesized by free radical polymerization. The well-established oligomer design involved the polymerization of hydrophobic domains of LA or/and SA, hydrophilic comonomers such as HEA or/and HEMA, and MA moieties as the reactive units. GPC confirmed the controlled molecular weights of the oligomers, while the comonomer composition of the ter-oligomers was affirmed by ^1^H-NMR studies. Incorporation of MA moieties and intactness were assessed by conductometric and Brown–Fujimori titrations. Also, the hydrophilicity of the ter-oligomers was confirmed by dissolution studies. The hydrophilic and cytocompatible formulations were developed by crosslinking gelatin molecules and anhydride units (amine–anhydride conjugation) in the presence of base molecules. The subsequent hydrogels revealed stiffness properties with low to high storage modulus values as evaluated by rheological studies. hADCs were successfully encapsulated in these hydrogels. The encapsulated cells remained viable and exhibited spreading and proliferation as time-dependent behavior. These oligomer-crosslinked hydrogels show potential for various biomedical and tissue engineering applications, especially in soft tissues. 

## 4. Materials and Methods

### 4.1. Chemicals

Lauryl acrylate (LA) and hydroxyethyl methacrylate (HEMA) were purchased from WPA Chemicals, Shanghai, China. 2-Hydroxyethyl acrylate (HEA), 2,2-azobis (2-methylpropionitrile, azobisisobutyronitrile/AIBN), gelatin powder (300B, porcine skin), stearyl acrylate (SA), and maleic anhydride (MA) were received from Glentham Life Sciences Ltd., Corsham, UK. Tetrahydrofuran (THF), diethyl ether, and dichloromethane were received from VWR Chemicals, BDH, Radnor, PA, USA. Deionized water was obtained from Quality Operations Laboratory (UVAS). NaOH (sodium hydroxide) and aniline (aminobenzene) was obtained from Merck (Darmstadt, Germany). Absolute ethanol (96% *v/v*), n-hexane, acetone, thymol blue, deuteroform (CDCl_3_), deuterated DMSO, and triethanolamine (TEOA) was received from Sigma-Aldrich (Burlington, MA, USA). Phosphate-buffered saline (PBS) tablets were received from Bio World (Bio-Plus Chemicals, Kuala Lumpur, Malaysia). 

### 4.2. Synthesis

Two novel sets of oligomers, i.e., *oligo* LA-*co*-HEA-*co*-MA (oLHM) and *oligo* LA-*co*-HEMA-*co*-MA (oLHeM), and *oligo* SA-*co*-HEA-*co*-MA (oSHM) and *oligo* SA-*co*-HEMA-*co*-MA (oSHeM), were synthesized using different combinations of hydrophobic domain-containing lauryl acrylate (LA) and stearyl acrylate (SA) with hydroxyethyl acrylate (HEA), hydroxyethyl methacrylate (HEMA), and reactive monomer (maleic anhydride–MA) under an inert N_2_ atmosphere at 60 °C for 18 h [19]. Free radical polymerization of the hydrophilic comonomers (HEA or HEMA) was initiated with the addition of 3% (N/N) AIBN dissolved in THF (Scheme 1). The ratio of comonomer (LA or SA): sum of HEA/HEMA and MA monomers was maintained as 1:20. The synthesized oligomers were purified using n-hexane three times and stored under reduced pressure.

### 4.3. Oligomer Characterization

#### 4.3.1. Titration

Conductometric titrations were performed to quantify the incorporated MA while the intact anhydride moieties were calculated by Brown–Fujimori Titration (BFT). The percentage of chemical anhydride intactness from conductometric titration (CT) and Brown–Fujimori titration (BFT) was calculated as:

$$Percentage\;intact\left[Anhydride\right]=\left[1-\frac{\left(\left[MA\right]BFT-\left[MA\right]CT\right)}{\left[MA\right]CT}\right]\times 100$$

#### 4.3.2. Gel Permeation Chromatography (GPC)

Molecular weights of the oligomers were measured using the GPC system (Agilent Technology, Santa Clara, CA, USA) and were calculated in comparison to polystyrene standards (PSS). The molecular weights were stated as number average molecular weight (M_n_), weight average molecular weight (M_w_), and dispersity value (Ð) [8]. 

#### 4.3.3. Nuclear Magnetic Resonance (NMR)

Oligomer solutions for ^1^H-NMR (20 mg/mL) and ^13^C-NMR (50 mg/mL) were analyzed in deuterated dimethyl sulfoxide (d-DMSO) using an NMR spectrometer (Bruker Biospin GmbH, Ettlingen, Germany) at 400 MHz and 25 °C. The post-acquisition data (1D spectrum after 16 scans) were analyzed using the Topspin software package (version 4.0.9). Chemical shifts (δ) were stated as parts per million (ppm) [8]. 

The combined data from ^1^H-NMR and the titrations were used to calculate the molar comonomer ratios in the synthesized oligomers. Integral I_1_ (Figure 1) was normalized to the number of protons in SA (in oSXM oligomers) and LA (in oLXM oligomers) and was used as an internal standard. Integral I_2_ refers to the protons of MA and could not be utilized to determine MA ratios unambiguously due to overlapping peaks in the NMR spectrum. Instead, the results of conductometric titrations were used to determine the MA content of the oligomers. Integral I_3_ was used to elucidate the numbers of moles of HEA or HEMA in the oligomers. 

#### 4.3.4. Oligomer Dissolution Characteristics

Oligomer–water interaction was determined by a previously published dissolution setup in deionized water [8,35]. A 10% oligomer sample was mixed with 1 mL of deionized water in a cuvette and was stirred continuously at 700 rpm on a magnetic stirrer (DLAB, Beijing, China) (Figure S1). The cuvette opening was kept covered with Parafilm^®^ to avoid any possible evaporation. Analysis was performed using a spectrophotometer (Cecil 7400s 7000 series UV-Vis Spectrophotometer) with de-ionized water used as the blank. The results were reported as percentage transmission–time graphs and time required to reach 60% of maximum transmission values.

#### 4.3.5. Hydrogel Fabrication 

Hydrogels were fabricated by mixing defined volumes of oligomer solution (20% *w/v* in DMSO), triethanolamine (50% *v/v* aqueous solution), and gelatin (4% *w/v* aqueous solution) (Figure S2, Table S1). 

#### 4.3.6. Rheological Characterization 

A controlled stress rheometer AR 1500 EX (TA Instruments, New Castle, DE, USA) was used to perform the rheological characterization. The gelation kinetics of the gel-forming constituents were analyzed using a 80 mm plate, 1° cone, and angular frequency of 1–25 Hz. The results were expressed as storage modulus values and loss modulus–time profiles. The pre-fabricated hydrogels, soaked overnight with PBS, were analyzed using frequency sweep tests. The results were reported as storage modulus (G′), loss modulus (G″), and complex viscosity (η). All experiments were performed in quadruplicate at 20 °C [8]. 

#### 4.3.7. In-Vitro Gel Degradation

The hydrogels were prepared as previously described and incubated in PBS preserved with 0.02% *w/v* sodium azide at room temperature. At pre-defined time intervals, the hydrogels were removed, dried in an oven, and the dry weights were measured. The buffer solution was changed on days 0, 2, 4, 6, 8, 10, 12, and 14. Analysis was performed in triplicate.

#### 4.3.8. In Vitro Cell Experiments

Cell culture: Adipose tissue-derived stromal cells (ADCs) were used to investigate the cytocompatibility of the oligomer-crosslinked hydrogels. Low glucose Dulbecco’s Modified Eagle Medium (DMEM-LG) with 10% fetal bovine serum (FBS) and 1% penicillin and streptomycin (P/S) was used as the cell culture medium. Cells were grown in 75 cm^2^ cell culture flasks at 37 °C in a 5% CO_2_ incubator and harvested upon attaining 80% confluency. ADCs were detached from the culture flasks using trypsin and 10,000 cells/well were seeded into 48-well plates with 500 µL of culture medium and used as the control group. For the fabrication of cell-encapsulated hydrogels, 100,000 cells were added in gelatin solution (prepared in DMEM) and mixed with oligomer solution in polypropylene molds. These molds were then transferred to 48-well plates and incubated with 500 µL of the culture medium. 

Cell viability: The influence of the oligomer-crosslinked hydrogels on the ADCs was investigated by WST assay on days 1, 3, and 7. The cell culture medium was exchanged on alternate days. For analysis, WST-1 reagent was mixed with the culture medium (1:10), the mixture was incubated at 37 °C for 1 h, and the absorbance was measured at 440 nm. Experiments were performed as quadruplicate and values were presented as the number of viable cells.

#### 4.3.9. Statistical Analysis

OriginPro 8.5.0 SRI was used to create the graphs and Chemdraw 16.0.0.82 was used to draw the chemical structures. For statistical analysis, independent two-sample *t*-test or ANOVA (one-way analysis of variance) was used. The independent two-sample *t*-test was used to compare two oligomer groups, while one-way ANOVA with post hoc Tukey’s test was used to compare the reactivity, molecular weights, and rheological factors of different groups varying in MA content.

## Figures and Tables

**Table 1:** Ratios of oligomer parts taken for *oligo* LA-*co*-HEMA-*co*-MA and *oligo* LA-*co*-HEA-*co*-MA.

General Name	Oligomer Name	Lauryl Acrylate (LA)	Hydroxyethyl Acrylate (HEA)	Hydroxyethyl Methacrylate (HEMA)	Maleic Anhydride (MA)
			Ratio: 1: 20 (LA: HEA/HEMA + MA)		
oLHMn	oLHM2.5	1	17.5	-	2.5
	oLHM5	1	15.0	-	5.0
	oLHM10	1	10.0	-	10.0
oLHeMn	oLHeM2.5	1	-	17.5	2.5
	oLHeM5	1	-	15.0	5.0
	oLHeM10	1	-	10.0	10.0

**Table 2:** Ratios of oligomer parts taken for *oligo* SA-*co*-HEMA-*co*-MA and *oligo* SA-*co*-HEA-*co*-MA.

General Name	Oligomer Name	Stearyl Acrylate (SA)	Hydroxyethyl Acrylate (HEA)	Hydroxyethyl Methacrylate (HEMA)	Maleic Anhydride (MA)
			Ratio: 1: 20 (LA: HEA/HEMA + MA)		
oSHM	oSHM2.5	1	16	-	4
	oSHM5	1	15	-	5
	oSHM10	1	10	-	10
oSHeM	oSHeM2.5	1	-	17.5	2.5
	oSHeM5	1	-	16	4
	oSHeM10	1	-	10	10

**Table 3:** Comonomer actual composition (theoretical input) and comonomer ratios obtained from ^1^H-NMR results.

Ter-Oligomer Groups	Oligomer	Comonomers in the Feed			Hydrophilic Comonomer (HEA or HEMA)	MA
		HEA	HEMA	MA		
oLHM	oLHM_2.5_	17.5	---	2.5	1.15	1.12
	oLHM_5_	15	---	5	1.24	1.23
	oLHM_10_	10	---	10	1.39	1.31
oLHeM	oLHeM_2.5_	---	17.5	2.5	1.08	1.10
	oLHeM_5_	---	15	5	1.13	1.13
	oLHeM_10_	---	10	10	1.16	1.28
oSHM	oSHM_2.5_	17.5	---	2.5	1.05	1.07
	oSHM_5_	15	---	5	1.11	1.13
	oSHM_10_	10	---	10	1.06	1.09
oSHeM	oSHeM_2.5_	---	17.5	2.5	1.08	1.02
	oSHeM_5_	---	15	5	1.12	1.04
	oSHeM_10_	---	10	10	1.07	1.07

**Table 4:** Molecular weight distributions for two sets of oligomers (oLXMs and oSXMs). Molecular weights have been represented as M_n_ (number average molecular weight), M_w_ (weight average molecular weight), and Ð (polydispersity index, M_w_/M_n_). All experiments were performed in triplicate.

Ter-Oligomer Groups	Oligomer	M_n_ (10^3^ Da)	SD	M_w_ (10^3^ Da)	SD	Ð (M_w_/M_n_)	SD
oLHM	oLHM2.5	2.39	0.21	3.42	0.17	1.45	0.16
	oLHM5	1.93	0.20	3.20	0.13	1.67	0.12
	oLHM10	1.81	0.14	4.54	0.08	2.53	0.16
oLHeM	oLHeM2.5	2.64	0.20	4.49	0.16	1.71	0.17
	oLHeM5	2.24	0.31	3.33	0.26	1.51	0.22
	oLHeM10	2.12	0.35	3.17	0.18	1.71	0.42
oSHM	oSHM2.5	3.61	0.26	4.90	0.29	1.36	0.02
	oSHM5	3.08	0.17	4.14	0.12	1.34	0.03
	oSHM10	2.41	0.42	3.69	0.20	1.53	0.28
oSHeM	oSHeM2.5	3.76	0.51	5.15	0.27	1.37	0.32
	oSHeM5	3.13	0.21	5.09	0.16	1.63	0.38
	oSHeM10	2.83	0.26	4.93	0.42	1.74	0.36

## Data Availability

Data are contained within the article.

## Supplementary Materials

The following supporting information can be downloaded at: https://www.mdpi.com/article/10.3390/gels9070564/s1, Figure S1: Dissolution setup for UV analysis to determine hydrophilicity; Figure S2: Hydrogel fabrication; Figure S3: ^13^C NMR Spectra of selected hydrophilic oligomers (a) oLHeM2.5, (b) oSHM-2.5; Table S1: Concentration of gel-forming components in stock solution and final gel.
