# Supplementary material for: Novel Hydrophilic Oligomer-Crosslinked Gelatin-Based Hydrogels for Biomedical Applications
Source: Gels. 2023 Jul 11;9(7):564. doi: 10.3390/gels9070564 (PMC10379017; doi:10.3390/gels9070564)
Supplement: Supplementary file 1 [file gels-09-00564-s001.zip › gels-2488461-supplementary.pdf]

Supplementary Data

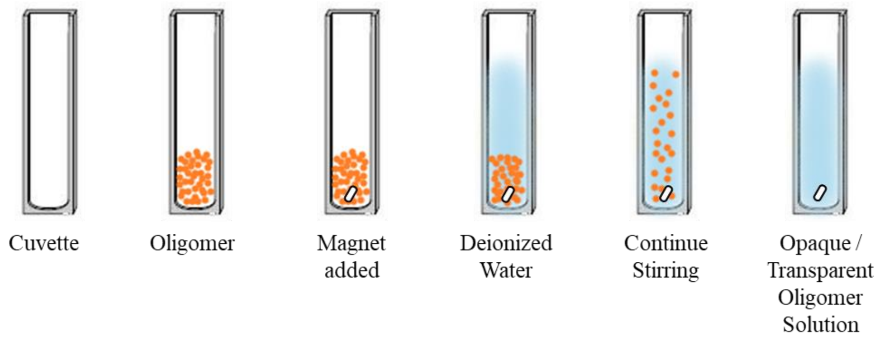

Figure S1. Dissolution Setup for UV Analysis to check Hydrophilicity.

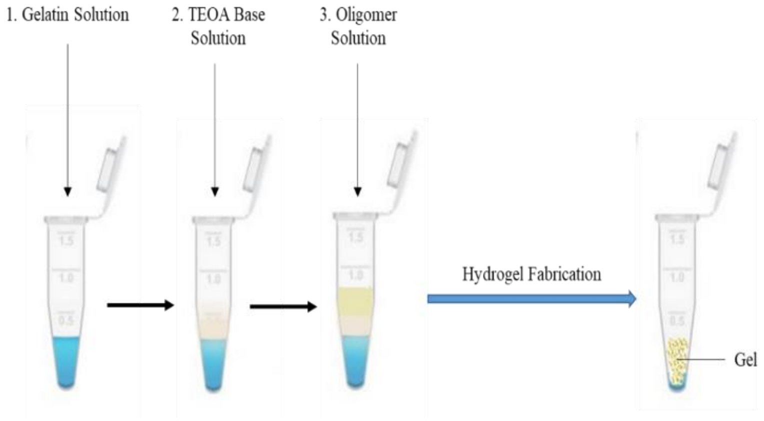

Figure S2. Hydrogel fabrication.

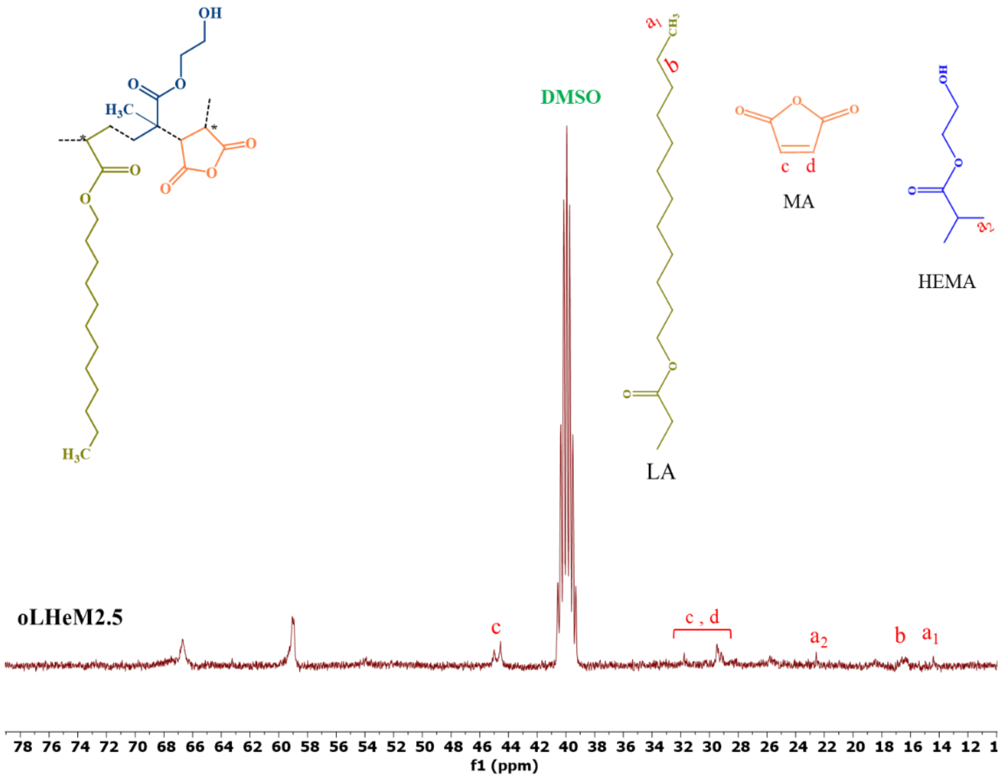

Figure S3 (a). ^{13}C NMR Spectra of selected hydrophilic oligomer (oLHeM2.5), having similar comonomer ratios as incorporated during synthesis. Spectral peaks are labelled with alphabetic letters that represent specific carbons of comonomers as shown in individual chemical structures.

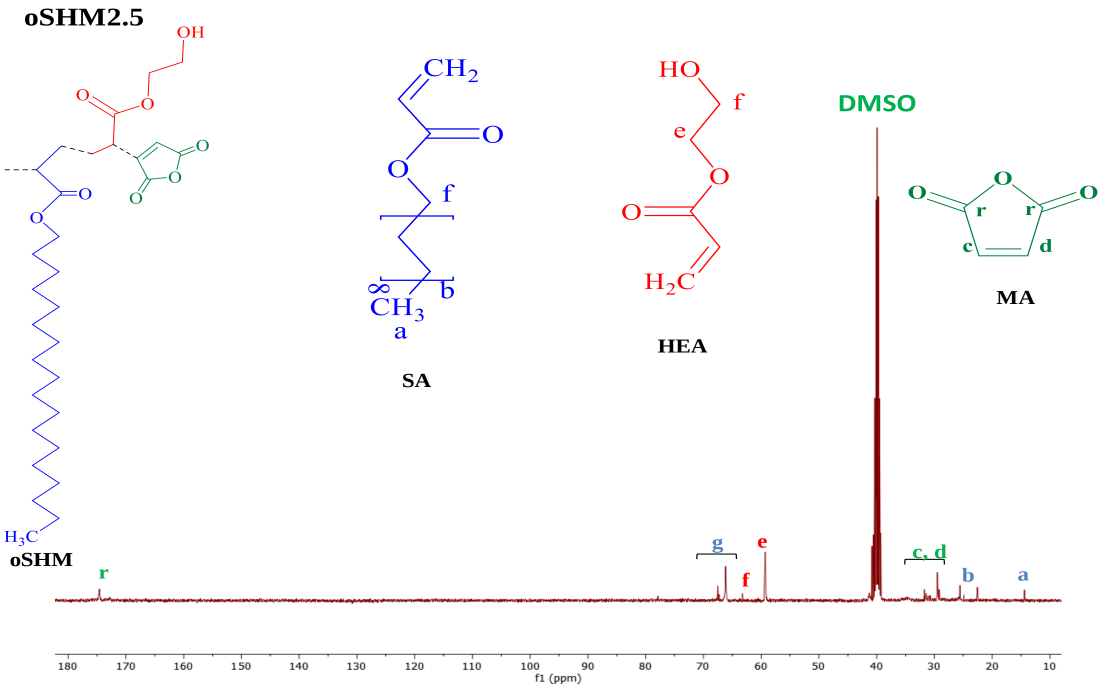

Figure S3 (b). Stacked ^{13}C-NMR spectra of selected oligomer: oSHM-2.5. The spectral peaks have labelled with letters that represent specific carbons in corresponding comonomers indicated in the chemical structures on top of spectra.

Table S1. Concentration of gel forming components in stock solution and final gel.

**Components of Hydrogel**	**Stock Solution**	**Volume**
	**%**	**uL**
Gelatin Solution	4	50
Base Solution	50	20
Oligomer Solution	20	30
